# The effect of disease-modifying anti-rheumatic drugs on skeletal muscle mass in rheumatoid arthritis patients: a systematic review with meta-analysis

**DOI:** 10.1186/s13075-022-02858-y

**Published:** 2022-07-19

**Authors:** Thales R. Hein, Leonardo Peterson, Barbara J. Bartikoski, Juliana Portes, Rafaela C. Espírito Santo, Ricardo M. Xavier

**Affiliations:** Universidade Federal do Rio Grande do Sul, Rheumatology, Rua Ramiro Barcelos, 2350, Porto Alegre, RS 90035-903 Brazil

**Keywords:** Systematic review, Rheumatoid arthritis, Sarcopenia, Muscle loss, Lean mass, Appendicular lean mass, Treatment, Drugs, DMARD

## Abstract

**Introduction:**

Rheumatoid arthritis (RA) is an autoimmune disease, characterized by chronic and systemic inflammation. Besides, it is known that RA patients may present several comorbidities, such as sarcopenia, a condition where patients present both muscle mass and muscle quality impairment. RA treatment is mostly pharmacological and consists in controlling systemic inflammation and disease activity. Despite that, the effect of pharmacological treatment on sarcopenia is not well characterized.

**Objective:**

To summarize the effects of disease-modifying anti-rheumatic drugs (DMARDs) on skeletal muscle tissue in rheumatoid arthritis (RA) patients.

**Methods:**

A systematic review of randomized clinical trials and observational studies was conducted using MEDLINE, Embase, Cochrane Library, and Web of Science. We selected studies with rheumatoid arthritis patients treated with disease-modifying anti-rheumatic drugs (DMARDs) that analyzed muscle mass parameters such as lean mass and appendicular lean mass. Methodological quality was assessed using the Newcastle-Ottawa Quality Assessment Scale. Standardized mean difference (SMD) and 95% confidence intervals (CI) were set. A meta-analysis of observational studies was performed using the R software, and we considered significant statistics when *p* < 0.05.

**Results:**

Nine studies were included in this systematic review. In the meta-analysis, DMARD treatment had no positive difference (*p* = 0.60) in lean mass. In the same way, in the appendicular lean mass parameter, our results showed that DMARDs did not have changes between baseline and post-treatment analysis (*p* = 0.93).

**Conclusion:**

There is no evidence of a significant effect of DMARD therapy, either synthetic or biological, on muscle mass. However, this association should be investigated with more studies.

## Introduction

Rheumatoid arthritis (RA) is a chronic, autoimmune disease characterized by systemic inflammation that affects mainly the joints [1]. In addition, RA leads to several comorbidities, such as cardiovascular disease and metabolic syndrome [2, 3]. Furthermore, RA patients are often associated with changes in body composition [3, 4] such as reduced skeletal muscle mass [5], decreased muscle strength [6], and poor physical function [4, 5, 7, 8]. The alterations in RA patients regarding body composition can be a description of a sarcopenic patient that carries the risk of physical incapacity, low quality of life, and death [9, 10]. The prevalence of sarcopenia ranges from 25.9 to 43.3% between cohort studies, a wide variation due to the differences in sample, age, gender, race, and definitions and methods of diagnosing sarcopenia [9, 11–13].

Muscle impairment in RA and during sarcopenia is associated with several mechanisms triggered by inflammatory signaling [14]. Inflammatory mediators, such as tumor necrosis factor α (TNF-α) and interleukin 1β (IL-1β), are pointed out as triggers of catabolic effects in muscle tissue [14]. Thus, interleukin 6 (IL-6) has a role in driving catabolism in muscle mass and anabolism in fat mass [15, 16].

The available treatments for RA aim to attenuate disease activity by blocking inflammatory mediators and their signaling or inducing anti-inflammatory and regulatory pathways [17]. Disease-modifying anti-rheumatic drugs (DMARDs) significantly improve disease activity and prevent joint damage in RA by targeting the key inflammatory pathways [17, 18]. The classification of therapeutic drugs are as follows: conventional synthetic DMARDs (csDMARDs), which comprehend methotrexate, leflunomide, sulfasalazine, and hydroxychloroquine; biological DMARDs (bDMARDs); and targeted synthetic DMARDs (tsDMARDs) [19, 20].

bDMARDs include targeting monoclonal antibodies against TNF (infliximab, adalimumab, certolizumab, and golimumab) and IL-6 (tocilizumab and sarilumab), soluble receptor for TNF (etanercept), an inhibitor of T-cell co-stimulation (abatacept), and anti-CD20 B-cell depleting monoclonal antibody (rituximab) [21]. The tsDMARDs are inhibitors of the Janus tyrosine kinase family (JAK), which targets intracellular signaling of type I and II cytokines (tofacitinib, baricitinib, upadacitinib, and filgotinib). Thus, tsDMARD effects are T cell reduction and decreased leukocyte recruitment to joint, resulting in less synovial inflammation and prevented joint damage in RA patients [22, 23]. In a recent narrative review published by our group [8], we found that csDMARDs, tsDMARDs, and bDMARDS have no benefits on muscle mass when used to treat RA patients. However, tocilizumab, an IL-6 inhibitor, may improve muscle mass by increasing appendicular lean mass and total lean mass in RA patients [8]. Also, glucocorticoids (CG) that can control disease activity in RA have known negative effects on the skeletal muscle in RA patients [24, 25]. Targeting inflammatory cytokines seems to have a positive role in muscle wasting. Data from many studies have shown that cytokine inhibition has been effective at preventing or treating muscle wasting. TNF-α blockade can partially revert muscle atrophy by suppression of the NF-κB pathway in several animal models and can prevent survival in aging mice [26–28]. Additionally, IL-6 have been independently implicated in some forms of muscle atrophy, and its deficiency attenuates atrophy in sepsis, diabetes melitus, and Duchenne muscular dystrophy [16, 29–31].

Nevertheless, sDMARDs and bDMARDs can control disease activity by blocking inflammatory signaling, but their effect on skeletal muscle tissue in RA patients remains unclear. Thus, this systematic review aims to summarize the current evidence on the effect of pharmacological treatment on skeletal muscle tissue in RA patients.

## Materials and methods

We conducted this systematic review in accordance with the PRISMA [32] guidelines after registering the protocol with the PROSPERO platform (CRD42021279386).

### PICOS/PECOS format

This systematic review with meta-analysis was based on a focused question described in a PICO/PECO format [33]. We established the following: Patient/Problem/Population= Rheumatoid arthritis patients, Intervention/Exposure= Chronic treatment with biological and synthetic DMARD and glucocorticoids, Comparison= Baseline and post treatment, Outcomes= Muscle mass parameters, such as muscle mass, fat-free mass, appendicular lean mass and lean mass; and Study= Randomized clinical trials and Observational studies.

### Data sources

The electronic databases used were Cochrane Library, PubMed, Embase, and Web of Science (DATA). We used a comprehensive search strategy tailored to each database. We contacted the authors, when necessary, for more information on the statistical methodology of the articles chosen as a reference. However, in some cases, we have not received any feedback.

### Search terms

Keywords and medical subject headings (MeSH) for the following terms,: “Rheumatoid arthritis,”, “Antirheumatic agents,”, “Methotrexate,”, “Leflunomide,”, “Sulfonamides,”, “Hydroxychloroquine,”, “Glucocorticoids,”, “Tumor necrosis factor,”, “Interleukin-6,”, “Janus Kinases,”, “Muscle,”, “Skeletal,”, “Body composition,”, “Cachexia,”, “Sarcopenia,” and related terms were selected. The term OR was used for Union of MeSH terms and "“entry terms",” and the term AND was used to attach the terms. The cComplete search is available below.

(Arthritis Rheumatoid [mh] OR Rheumatoid Arthritis [all fields] OR RA [all fields]) AND (Antirheumatic Agents [mh] OR Antirheumatic*[all fields] OR Anti-rheumatic*[all fields] OR DMARD [all fields] OR Methotrexate [mh] OR Methotrexate [all fields] OR Leflunomide [mh] OR Leflunomide [all fields] OR Sulfonamides [mh] OR Abatacept [all fields] OR rituximab [all fields] OR Sulfonamides [all fields] OR Hydroxychloroquine [mh] OR Hydroxychloroquine [all fields] OR Glucocorticoids [mh] OR Glucocorticoid*[all fields] OR Tumor Necrosis Factor-alpha [mh] OR Tumor Necrosis Factor-alpha [all fields] OR TNFalpha [all fields] OR TNF-alpha [all fields] OR Interleukin-6[mh] OR Interleukin-6[all fields] OR Janus Kinases [mh] OR Janus Kinases [all fields]) AND (Muscle Skeletal [mh] OR Muscle mass [all fields] OR Body Composition [mh] OR Body Composition [all fields] OR Cachexia [mh] OR Cachexia [all fields] OR Sarcopenia [mh] OR Sarcopenia [all fields])

### Inclusion/exclusion criteria

Randomized clinical trials and observational studies with patients diagnosed with RA that were treated with bDMARD, tsDMARD, and csDMARD that analyzed muscular parameters, and articles that were written in the English language were included. No restrictions about the year of the studies were applied. Articles that reported data from RA patients < 18 years old, clinical trials, experimental studies, reviews, meta-analyses, studies of patients without RA, and studies that proposed acute treatment were excluded.

### Study selection and data extraction

Title, abstract, and full-text screening were performed in pairs by two independent reviewers (Hein, TR, and Bartikoski, BJ). The reviewers extracted the data from the studies independently, using a pre-established data sheet, which is available upon request. All data from the study were screened using a bibliographic management program (Mendeley®, version1.17.9). Disagreements about data abstraction were resolved by a discussion between the two reviewers. If no agreement could be reached, a third reviewer (Santo, RCE) provided the final decision. The information extracted during the data abstraction included authors’ names, date of publication, journal of publication, number of participants in the study, the age group of the population, type of population, type of treatment, duration of treatment, treatment posology, and results obtained for lean mass and appendicular lean mass. After the authors’ agreement, nine studies were included in this review. The baseline mean and after-treatment mean were extracted and converted and the delta of the mean (difference of final mean and baseline mean) for meta-analysis. In one study [34], we estimated the baseline mean from graph bars with the ImageJ software.

### Methodological quality assessment

Methodological quality was assessed by the Newcastle-Ottawa Scale for cohort studies or for randomized clinical trials [35–37] by two independent reviewers (Santos, LP, and Portes, JKS). In these scales, each study was judged by questions about groups of criteria: selection of cohort, comparability of the study, and ascertainment of the outcomes for cohort studies and selection, comparability, and exposure for randomized clinical trials. For each item, in the selection, outcome, and exposure groups, a maximum of one star can be assigned, and for the comparability group, a maximum of two stars can be assigned. So, the maximum possible score was 9 stars. Based on the scale, studies with scores of 3 or 4 in the selection, 1 or 2 scores in comparability, and 2 or 3 in outcome or exposure were classified as good quality studies. On the other hand, studies with 2 stars in the selection, 1 or 2 stars in comparability, and 2 or 3 stars in outcome and exposure were classified as fair-quality studies. Finally, studies with scores of 0 or 1 in the selection, 0 scores in comparability, or 0 or 1 score in outcome or exposure were classified as poor-quality studies.

### Risk of bias assessment

The risk of bias in the randomized clinical trials was assessed using the Risk of Bias Tool 2.0 (RoB2) from Cochrane to randomized clinical trials [38]. The evaluators examined the randomization process, deviations from intended interventions, missing outcome data, measurement of the outcome, and selection of the reported results. Thus, the studies were classified into low, moderate, or high risk of bias.

### Statistical analysis

The meta-analysis was conducted using the mean_change_ and SD_change_ from each study. All outcome measures were continuous variables. A meta-analysis, representing the effects of interventions, was performed: the random-effects model with the mean difference (MD) MD was performed when studies reported outcomes using the same assessment scale or assessment instrument.

The 95% confidence intervals (CI) were used, and the heterogeneity of the studies included in the meta-analysis was assessed using the inconsistency test (*I*^2^). We considered low, moderate, and high inconsistency in the approximated values of 25%, 50%, and 75%, respectively [39, 40]. The software used for statistical analysis was RevMan (Review Manager 5.4.1, The Cochrane Collaboration, 2020), and we considered it significant statistically when *p* < 0.05.

## Results

### Search strategy

**Fig. 1:**
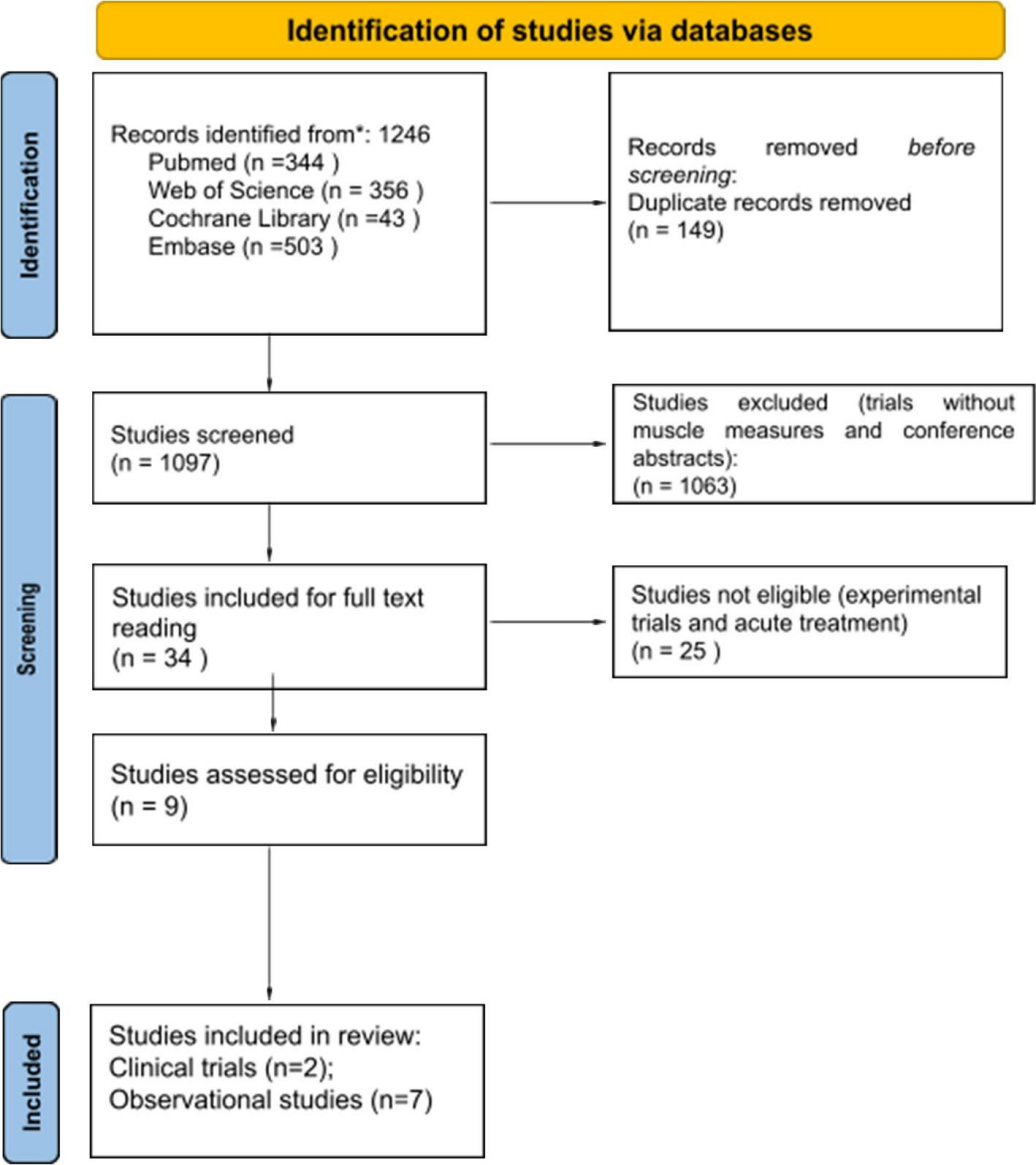
Search and inclusion/exclusion criteria

We identified 1123 possible studies (134 duplicate publications) based on our search items. First, the title and abstract of the 1244 studies were screened. After this process, 32 articles were included for the full-text screening. Finally, after the full-text reading, we included nine studies: Engvall et al. [41] and Marcora et al. [42] as randomized clinical trials and Al Khayyad et al. [43], Vial et al. [44], Tournadre et al. [45], Toussirot et al. [46], Ferraz-Amaro et al. [47], Metsios et al. [48], and Chikugo et al. [34] as observational studies. The search and inclusion/exclusion criteria are described in Fig. 1.

### Characteristics of the studies

Of the nine studies included, four of them were performed in France [42, 44–46], one of them in the UK [48], one in Spain [47], one in Japan [34], one in Sweden [41], and one in Italy [43]. Studies included were published between 2007 and 2020. Only one study was performed using female patients [34] while the other four were performed with male and female patients [45–48]. Included papers reported sample size ranged from 8 to 146 subjects, patients’ age means from 50 to 61 years. Studies also showed baseline DAS-28 ranged from 3.0 to 6.1 [34, 45–48]. Characteristics of the included studies are described in Tables 1 and 2.

**Table 1:** Characteristics of the observational studies included in the systematic review with meta-analysis

First author name	Country	Gender	Year	Sample size	Age	Treatment	Dose	Measure	Lean mass (kg)	App. lean mass (kg)	Method of BC assay	Pre-DAS-28	Post-DAS-28
Tournadre [23]	France	M/F	2017	21	57.8 ± 10.5	Tocilizumab 12 months	NI	Lean mass App. lean mass	Baseline: 42.1 (± 11.1) Final: 43.2 (±11.3)	Baseline: 17.7 (± 5.4) Final: 18.7 (± 5.6)	DEXA	4.94 ± 1.25	2.8 ± 1.5
Toussirot [22]	France	M/F	2020	107	56.6 ± 13.5	Tocilizumab 12 months	8 mg/kg (monthly)	Lean mass	Baseline: 40.76 (± 8.4) Final: 42.11 (± 8.9)	–	DEXA	4.93 ± 1.3	2.3 ± 1.3
Ferraz-Amaro [24]	Spain	M/F	2011	16	50.8 ± 14.6	Anti-TNF 12 months	Varied	Lean mass	Baseline: 53.7 (NI) Final: 50.5 (NI)	–	BIA	5.58 ± 0.87	2.89 ± 1.37
Metsios [25]	United Kingdom	M/F	2007	20	61.1 ± 6.8	Anti-TNF 3 months	NI	Lean mass	Baseline: 50.9 (± 12.7) Final: 51.1 (± 12.5)	–	BIA	5.66 ± 0.7	3.59 ± 0.7
Chikugo [26]	Japan	F	2018	4	55.3 ± 19.5	Tofacitinib 6 months	NI	App. lean mass	–	Baseline: NI Final: 20.4 (± 4.0)	BIA	5.1 ± 0.8	NI
Al Khayyat [27]	Italy	F	2021	20	65 ± 12.9	Rituximab 18 months	Eight infusions of 500 mg~1 g	Lean mass App. lean mass	Baseline: 39.94 ± 8.74 Final: 38.64 ± 8.19	Baseline: 16.21 ± 3.60 Final: 17.84 ± 4.03	DEXA	NI	NI
Vial [28]	France	Male/Female	2021	83	58.5 ± 10.8	Biologic DMARD (TNFi and non-TNFi)	NI	Lean mass	TNFi Baseline: 49.6 ± 10.8 Final: 50.7 ± 11.3 Non TNFi Baseline: 47.7 ± 11.0 Final: 47.4 ± 10.9	–	DEXA	4.21 ± 1.1	NI

*BIA* bioimpedance, *DEXA* dual-energy X-ray absorptiometry, *NI* not informed, *TNF* tumor necrosis factor, *TNFi* TNF inhibitor, *BC* body composition, *M* male, *F* female

**Table 2:** Characteristics of the clinical trials included in the systematic review with meta-analysis

First author name	Country	Gender	Year	Sample size	Treatment	Mean age	Dose	Measure	Method of BC assay	Pre-DAS-28	Post-DAS-28
Marcora [29]	France	M/F	2006	24	Group 1: etanercept Group 2: MTX	NI	Group 1: 50 mg/week Group 2: 7.5~15 mg/week	Lean mass	DEXA	Group 1: 6.1 ± 0.7 Group 2: 5.8 ± 1.1	Group 1: t3.2 ± 1.5 Group 2: 3.1 ± 1.5
Engvall [30]	Sweden	M/F	2010	40	Group 1: MTX + SSZ + HCQ Group 2: infliximab + MTX	Group 1: 59.5 Group 2: 56.0	Group 1: 20 mg/week MTX + 2000 mg/day SSZ + 400 mg/day HCQ Group 2: 20 mg/week MTX + 3 mg/kg infliximab (weeks 0, 2, 6 and every 8 weeks)	Lean mass App. lean mass	DEXA	Group 1: 4.3 Group 2: 4.8	NI

*BIA* bioimpedance, *DEXA* dual-energy X-ray absorptiometry, *NI* not informed, *TNF* tumor necrosis factor, *BC* body composition, *M* male, *F* female, *MTX* methotrexate, *SSZ* sulfasalazine, *HCQ* hydroxychloroquine

### Characteristics of treatments

Among the nine papers included, the treatments used in the studies were tocilizumab [45, 46], anti-TNF [47, 48], JAKi [34], rituximab [43], bDMARDs [44], etanercept [42], methotrexate [41, 42], sulfasalazine [41], and hydroxychloroquine [41].

### Methods of assessment of the muscle mass and treatment effect

Three of nine studies (33%) used bioimpedance as a measurement method [34, 47, 48], while the other six (66%) used dual-energy X-ray absorptiometry (DEXA) [41–46]. Despite being different methods of assessing muscle mass, studies have shown that these methods have good validity and agreement [49, 50]. Toussirot et al. used lean mass, and the proposed treatment showed significant improvement in this parameter (3.3%) [46]. Tournadre et al. analyzed parameters lean mass and appendicular lean mass showing significant benefits in both parameters after one year of treatment with tocilizumab (2.6% in lean mass and 5.6% in appendicular lean mass) [45]. Ferraz-Amaro et al. (5.9%) and Metsios et al. (0.39%) used lean mass as a parameter, but both showed no significant improvement after anti-TNF treatment [47, 48]. Chikugo et al. used appendicular lean mass as a parameter and showed no significant changes in this parameter after JAKi treatment (0.49%) [34]. Al Khayyat et al. [43] used both lean mass and appendicular lean mass as parameters and showed a decrease (3.3%) in lean mass and an increase (10%) in appendicular lean mass. Vial el al [44]. used lean mass as a parameter and showed an improvement of lean mass in the TNFi group (2.2%) and a decrease (0.7%) of lean mass in the non-TNFi group.

### Methodological quality of the studies

In the methodological quality assessment of the nine studies, eight studies [34, 41–45, 47, 48] were classified as good-quality studies, and one was classified as a poor-quality study [46]. Data were described in Table 3.

**Table 3:** Methodological quality of the studies

Author	Year	Cohort selection				Comparability			Outcome ascertainment			Total score	Quality
Article		1	2	3	4	1a	1b	1c	1	2	3		
Chikugo M et al.	2018	-	★	★	★	★			★	★	★	7	Good quality
Ferraz Amaro et al.	2011	★	★	★	★	★★			★	★	No	8	Good quality
Metsios et al.	2007	★	★	★	★	★			★	★	No	7	Good quality
Tournadre et al.	2017	★	-	★	★	★			★	★	No	6	Good quality
Toussirot et al.	2020	★	-	★	★	-			★	★	No	5	Poor quality
Al Khayyat et al.	2021	★	-	★	★	-			★	★	★	6	Good quality
Vial et al.	2021	★	★	★	★	★★			★	★	No	8	Good quality
Engvall et al.	2010	★	★	★	-	★★			★	★	No	7	Good quality
Marcora et al.	2006	★	★	★	-	-			★	★	★	6	Good quality

### Risk of bias of studies

In the risk of bias analysis, two of the two studies [41, 42] were classified with a high risk of bias. Data was described in Table 4.

**Table 4:** Risk of bias analysis

Study ID	Randomization process	Deviations from intended interventions	Missing outcome data	Measurement of the outcome	Selection of the reported result	Overall
Engvall et al.	High	Some concerns	Low	Low	Low	**High**
Marcora et al.	High	Some concerns	Low	Low	Low	**High**

### Meta-analysis of lean mass

**Fig. 2:**
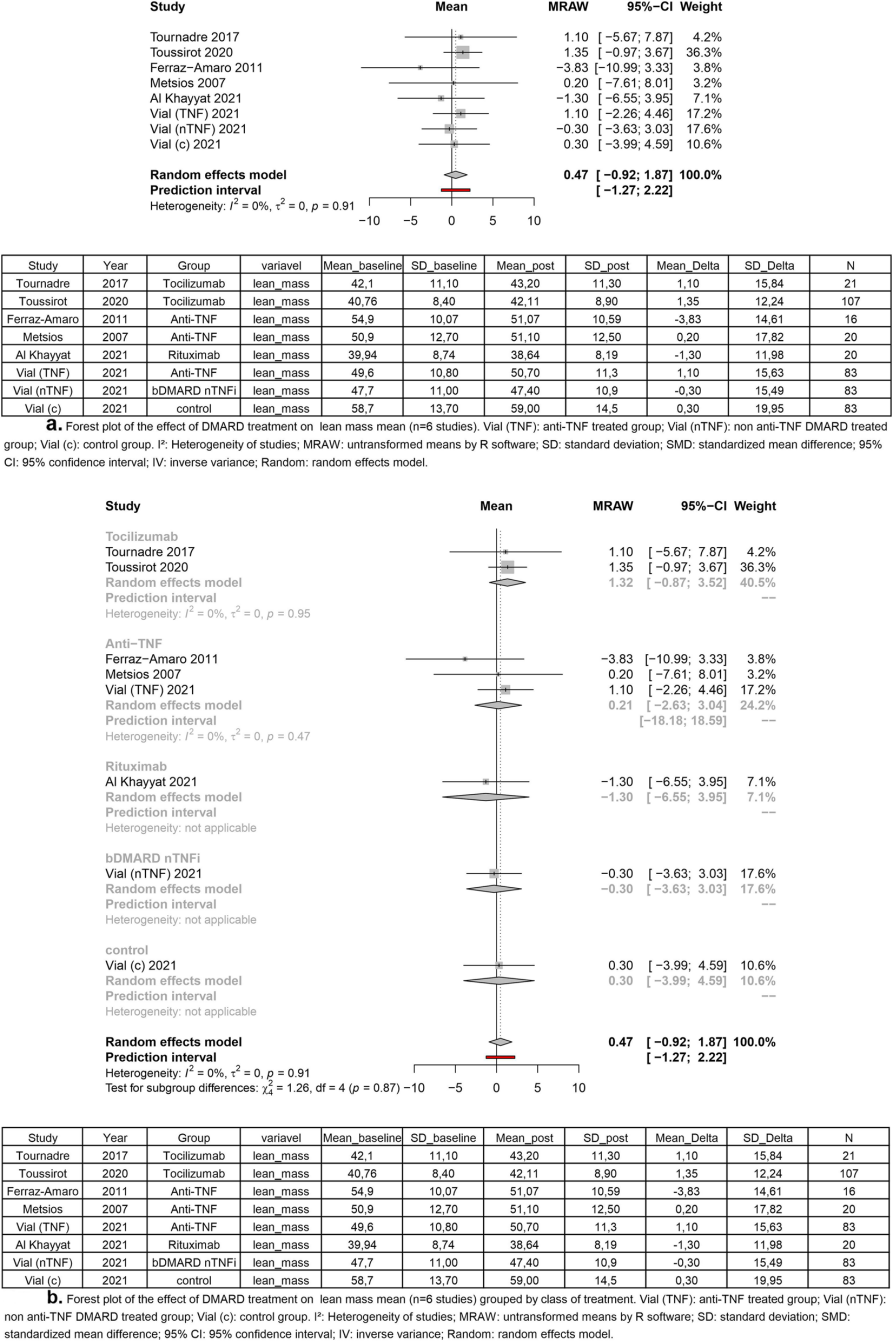
In general analysis, the treatment with DMARD was not able to increase lean mass in patients

**Fig. 3:**
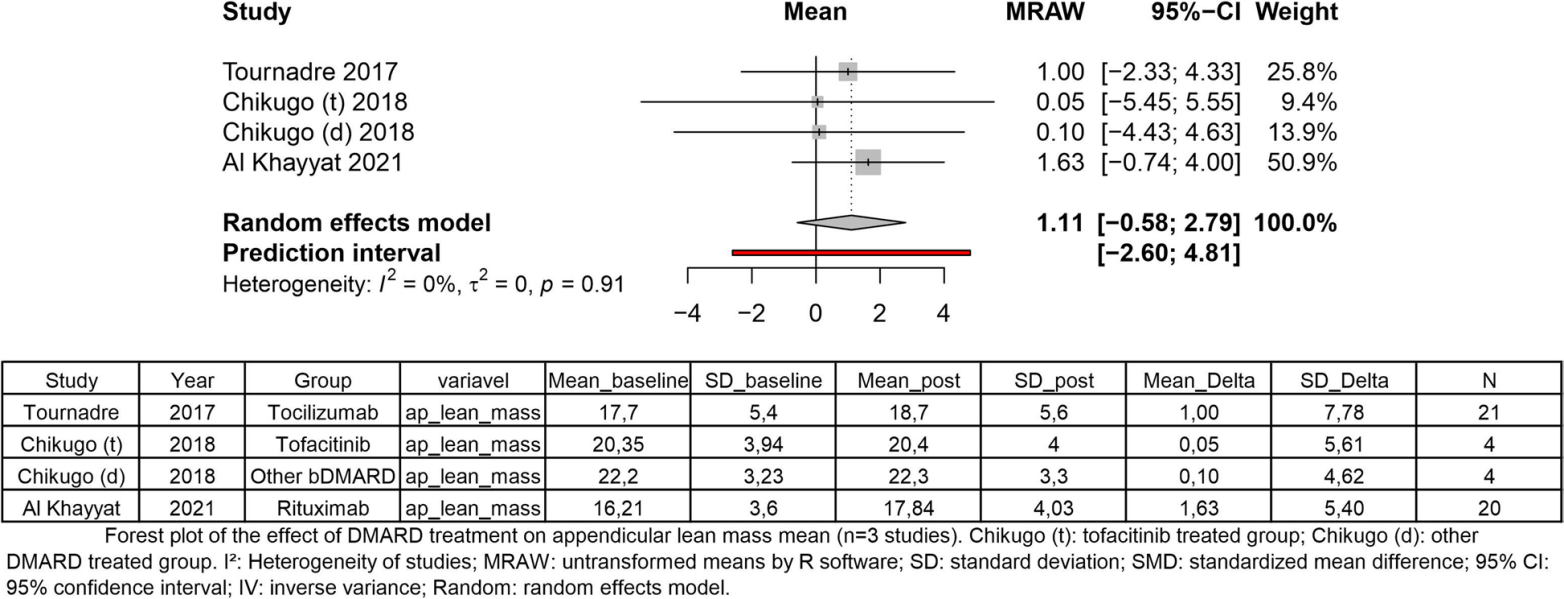
Positive mean of tocilizumab treatment and TNFi treatment

Four out of seven observational studies performed lean mass measures [45–48]. About this outcome, we performed two different methods in our meta-analysis: a general analysis comparing the four studies, and a subgroup analysis comparing types of treatment. Two studies used tocilizumab as treatment, and the other two used anti-TNF therapy. Despite the lack of significant difference, in the general analysis, five [44–46, 48] of eight groups analyzed have shown a positive delta of lean mass, and three [43, 44, 47] groups have shown a negative delta. In general analysis, the treatment with DMARD was not able to increase lean mass in patients (mean = 0.47; 95% CI [− 0.92 to 1.87]; *I*^2^, 0% *p* = 0.91) (Fig. 2). In the subgroup analysis, tocilizumab treatment (mean = 1.32; 95% CI [− 0.87 to 3.52;] *I*^2^, 0%; *p* = 0.95) and TNFi treatment (mean = 0.21; 95% CI [− 2.63 to 3.04;] *I*^2^, 0%; *p* = 0.47) had positive mean (Fig. 3). In rituximab (mean = − 1.30) and bDAMRD nTNFi (mean = − 0.30) treatment, the mean was negative.

### Meta-analysis of appendicular lean mass

**Fig. 4:**
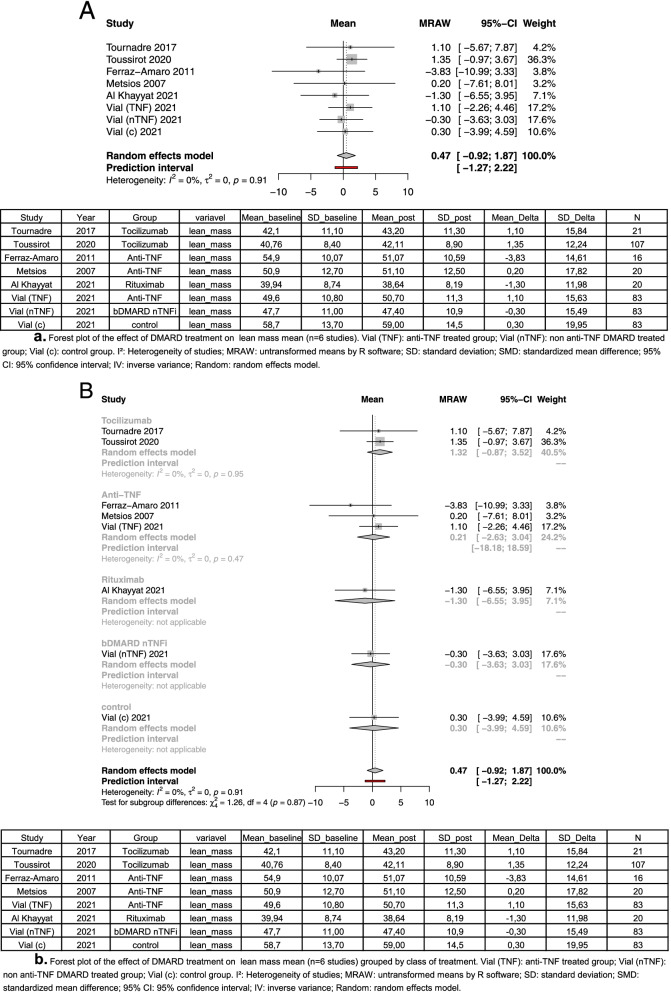
Regardless of the increase of the mean appendicular lean mass, treatment with DMARD showed no significant change in appendicular lean mass delta

Regarding appendicular lean mass, three studies have measured this outcome. Still, one of these studies has performed a trial with two groups of treatment: one group treated with tofacitinib, and one group treated with other bDMARDs [34]. Regardless of the increase of mean appendicular lean mass, treatment with DMARD showed no significant change in appendicular lean mass delta (mean = 1.11, 95% CI [− 0.58 to 2.79]; *I*^2^, 0%; *p* = 0.91) (Fig. 4).

## Discussion

As a result of this systematic review with meta-analysis, we found that DMARD treatment did not appear to induce significant muscle mass changes in RA patients. Still, regarding lean mass measurement, we described in subgroup analysis that anti-IL-6 and anti-TNF treatments were more related to the gain of lean mass than other DMARD therapies. Besides, considering the slight mass gain in both lean mass and appendicular lean mass and the small number of studies, we cannot exclude the possibility of a beneficial effect, particularly in anti-IL6 and anti-TNF therapy. This systematic review with meta-analysis is the first to verify the effect of DMARD treatment and its subclasses in muscle mass parameters.

Considering the muscle mass loss present in RA sarcopenia [10], DMARD treatment not only prevented this but also showed a trend of a slight gain of muscle mass in analyzed parameters. Dao et al. (2021) [51] investigated the associations between RA treatment and sarcopenia prevalence. Inherently, the authors showed that RA patients on csDMARD treatment had a lower prevalence of sarcopenia compared to RA patients on bDMARD. tsDMARD treatment had no association with sarcopenia. As we also saw in our review, Dao et al. emphasized the small number of papers in the literature and pointed out that it could be the reason for the lack of associations.

In our review, studies showed that IL-6 inhibition tended to be related to slight lean mass gain. IL-6 can bind the membrane IL-6 (IL-6R) and induce intracellular signals [52]. Still, IL-6 can also bind to soluble receptors (sIL-6R) creating a complex able to stimulate cells that do not have the membrane receptor [53]. Due to these mechanisms, the IL-6 effect in cells can be dualistic, being either inflammatory or anti-inflammatory [16]. Indeed, in an acute exercise setting, IL-6 secreted by muscle cells can drive muscle growth signaling, muscular regeneration, and activation of muscle stem cells [54]. On the other hand, chronic expression of IL-6 by inflammatory and immune cells is related to the induction of muscle atrophy and protein degradation [55, 56]. These effects occur by IL-6R binding leading to activation of the JAK/STAT complex [57] and signaling the increased expression of catabolic genes, such as muscle RING-finger protein-1 (MURF1), ubiquitin-proteasome subunits, caspases and cathepsins [14]. Thus, we consider that anti-IL6 therapy could have a positive effect on muscle mass in conditions of chronic inflammation based on its influence on important routes of inflammatory signaling and on its role as a locally secreted myokine [58]. Differently from other proinflammatory cytokines, which are mostly secreted by inflammatory cells and their action is generally systemic, IL-6 is secreted by muscle cells for paracrine communication leading to potent local signaling [59].

TNF-α is another key factor in muscle impairment in RA [60]. TNF-α inhibition therapy also seemed to have a positive effect on lean mass in AR patients. At a molecular level, TNF-α is the main responsible for the NFκꞵ activation pathway [61, 62], a transcript factor known to drive the subsequent expression of inflammatory mechanisms [63]. With the meta-analysis results, we speculate that despite its approved effect against RA disease activity, blocking systemic inflammation, anti-TNF treatment tended to have a local effect to block TNF downstream in the muscle being able to prevent AR muscle loss [64]. Interestingly, in both randomized clinical trials mentioned in our review, Marcora et al. and Engvall et al. did not present, in their results, significant change in both lean mass and appendicular lean mass, when patients were treated with anti-TNF drug [41, 42].

JAKi treatment, a more recent approach, has been demonstrated to be effective against RA inflammation [65]. The JAK/STAT pathway is known for acting together with cytokine receptors carrying the intracellular signaling through the phosphorylation of STATs [57, 66, 67]. For example, JAK/STATs are attached to IL-6 membrane receptors and are responsible for activating the transcription of inflammatory genes [68]. In our review, we showed that JAKi treatment did not present a significant effect on appendicular lean mass. Still, its effect was similar to DMARD treatment performed in the same study [34]. We believe that JAKi analysis was limited by the lack of studies and the study sample size.

In this review, we used Newcastle Ottawa to describe the quality of each study included in our systematic review with meta-analysis. The majority of studies were identified with good quality. Finally, this systematic review with meta-analysis has some limitations. First, there were a small number of studies included. Furthermore, the studies included were performed by enrolling both male and female patients, and it is known that men have higher muscle mass than women.

We conclude that DMARDs have no effect on muscle mass parameters in rheumatoid arthritis patients. Indeed, we showed that DMARD treatment was not able to have a positive effect both in lean mass (total lean mass including trunk) and appendicular lean mass (lean mass of arms and legs only), results that coincide with clinical trials available in the literature. However, this review could be a path to better understanding the treatment of RA muscle loss, being the first to systematically analyze the literature about it. We believe that the limitations found in our review, such as the small number of studies and sample size, may have been relevant for not having found differences in our analyses. Emphasizing this is important to drive and induce researchers to develop investigations about it. In addition, the enlightenment of how DMARDs act in muscle mass is important for the formulation of treatment protocols that can treat not only autoimmune and inflammatory diseases but also muscle-wasting conditions such as sarcopenia and cachexia. Finally, by summarizing and qualifying the data about the relationship between DMARDs and muscle mass in RA, this systematic review is crucial to enlighten the evidence presented in the literature.

## Conclusion

We conclude that this review was the first to summarize the data about the relationship between DMARDs and muscle mass. In addition, we have that DMARD treatment has no positive effect on rheumatoid arthritis muscle mass loss. With this review, we contribute to enlightenment in DMARD treatment in rheumatoid arthritis once it does not have any approved pharmacological therapy for comorbidities such as muscle loss.

## Data Availability

The authors declare that all data supporting the findings of this study are available within the article and its supplementary information files.

## Abbreviations

RA: Rheumatoid arthritis
MD: Mean difference
SMD: Standardized mean difference
CI: Confidence interval
DMARD: Disease-modifying anti-rheumatic drug
bDMARD: Biological disease-modifying anti-rheumatic drug
tsDMARD: Target synthetic disease-modifying anti-rheumatic drug
csDMARD: Conventional synthetic disease-modifying anti-rheumatic drug
TNF-α: Tumor necrosis factor-alpha
IL-6: Interleukin 6
sIL-6R: Soluble interleukin 6 receptor
IL-1β: Interleukin 1 beta
JAK: Janus kinase
IL-2: Interleukin 2
IL-7: Interleukin 7
DEXA: Dual-energy X-ray absorptiometry
BIA: Bioimpedance
MuRF1: Muscle RING-finger protein 1
TGF-β: Transforming growth factor-beta
JAK/STAT: Janus kinase/signal transducer and activator of transcription
MTX: Methotrexate
